# The Offspring of Blood Relations

**Published:** 1855-03

**Authors:** 


### THE OFFSPRING OF BLOOD-RELATIONS.

We beg leave to call the attention of our readers to the subjoined circular of Dr. BARTLETT, and request the favor of the editors of medical journals to copy it. That the evil which Dr. BARTLETT proposes to remedy is a momentous one no one can doubt who has enjoyed many opportunities for observation, and whether or not any legislative interference is probable or feasible, the discussion of the subject and the strong light that may be brought to bear upon it will unquestionably tend to abate the pernicious practice. Until statistics are accumulated few persons can be aware of the extent of the mischief flowing from such marriages, and from the amount of testimony already collected by Dr. BARTLETT, we have no doubt his researches will bring out impressive results.

### CIRCULAR.

My attention has recently been directed to the defects in the offspring of parents related by consanguinity. So frequent and serious have the ill-results of the intermarriage of blood-relations been found, that I deem it philanthropic to prepare a report on the subject, with a view to leading to legislative action on the subject. That my report may be as full and satisfactory as possible, I have to beg of physicians or others the favor of sending me histories of such cases as may have fallen under their observation.

The following questions, I believe, cover every point of interest in each case. To prevent confusion, the names of the parties, or their initials should be given, though, of course, these will be suppressed in the report:

How many instances of intermarriage among blood-relations have you known?

In how many of these were all the offspring perfect?

What was the state of the health of each parent? Had the mother borne children previously? if so, were the first children of her relative inferior to the latter ones?

Did the parents resemble one another? that is, had they the same family peculiarity of form, manner, mode of thought, &c.?

Have the parents, in any case, been themselves the offspring of blood-relations ?

How many children followed the union ? How many of them were idiotic, epileptic, rachitic, or deaf ? If none were so, what is the absolute and relative cleverness of each ?

In cases where the offspring have grown up, is there any tendency to insanity, epilepsy, or any similar disorder ?

Has the mother of imperfect offspring married again ? if so, what is the character of the children by this union ?

JOHN BARTLETT, M. D.

*Louisville*, *Ky.*, *March* 10, 1855.

